# LC–MS/MS Method for Measurement of Thiopurine Nucleotides (TN) in Erythrocytes and Association of TN Concentrations With TPMT Enzyme Activity

**DOI:** 10.3389/fphar.2022.836812

**Published:** 2022-03-21

**Authors:** Amol O. Bajaj, Mark M. Kushnir, Erik Kish-Trier, Rachel N. Law, Lauren M. Zuromski, Alejandro R. Molinelli, Gwendolyn A. McMillin, Kamisha L. Johnson-Davis

**Affiliations:** ^1^ ARUP Institute for Clinical and Experimental Pathology, Salt Lake City, UT, United States; ^2^ University of Utah Health Sciences Center, Department of Pathology, Salt Lake City, UT, United States; ^3^ Department of Pharmaceutical Sciences, St. Jude Children’s Research Hospital, Memphis, TN, United States

**Keywords:** 6-thioguanine, 6-methylmercaptopurine, mass spectromerty, thiopurine methyl transferase, clinical evaluation

## Abstract

Monitoring concentrations of thiopurine metabolites is used clinically to prevent adverse effects in patients on thiopurine drug therapy. We developed a LC–MS/MS method for the quantification of 6-thioguanine (6-TG) and 6-methylmercaptopurine (6-MMP) in red blood cells (RBCs). This method utilizes an automated cell washer for RBC separation from whole blood samples and washing of the separated RBCs. The lower limit of quantification of the method was 0.2 μmol/L for 6-TG (∼50 pmol/8 × 10^8^ RBC) and 4 μmol/L for 6-MMP (∼1,000 pmol/8 × 10^8^ RBC). The total imprecision of the assay was <3.0%. The upper limit of linearity for 6-TG and 6-MMP was 7.5 μmol/L and 150 μmol/L, respectively. The stability of the thiopurine metabolites under pre- and post-analytically relevant conditions was also evaluated. A good agreement was observed between this method and validated LC–MS/MS methods from three laboratories, except for ∼40% low bias for 6-MMP observed in one of the methods. The assessment of the association between 6-TG and 6-MMP concentrations with thiopurine S-methyltransferase (TPMT) phenotype and genotype demonstrated a statistically significant difference in the thiopurine metabolite concentrations between the TPMT groups with normal and intermediate activity of 6-MMP (*p* < 0.0001), while the difference in 6-TG concentrations was statistically not significant (*p* = 0.096). Among the samples with normal TPMT activity, higher concentrations of 6-MMP (*p* = 0.015) were observed in pediatric samples than in the samples of adults. No statistically significant differences were observed in the distributions of 6-TG and 6-MMP concentrations among the evaluated genotypes.

## Introduction

The thiopurine drugs azathioprine (AZA) and 6-mercaptopurine (6-MP) are anticancer and immunosuppressive drugs, which are used to treat patients with acute lymphoblastic leukemia and several autoimmune diseases, including the inflammatory bowel disease (Karran and Attard, 2008; Sandborn, 2009). AZA is a prodrug that is converted to 6-MP, which may then enter several pathways. Hypoxanthine guanine phosphoribosyl transferase (HPRT) and several other purine salvage pathway enzymes participate in the conversion of 6-MP into the active cytotoxic metabolites, 6-thioguanine nucleotides (6-TGN) (Supplementary Figure S1). Two pharmacologically inactive metabolites, 6-thiouric acid and 6-methylmercaptopurine (6-MMP), are produced through the action of the enzymes thiopurine S-methyltransferase (TPMT) and xanthine oxidase (XO), respectively (Swann et al., 1996; Gearry and Barclay, 2005). 6-MMP and related 6-methylmercaptopurine nucleotides (6-MMPN) are potentially hepatotoxic. In patients with very low TPMT activity, there is an increased risk of developing myelosuppression, caused by the increased levels of cytotoxic 6-TGNs (Evans et al., 2001; Schwab et al., 2002). Enzyme NUDT15 is involved in the dephosphorylation of 6-TGNs to inactive metabolites; individuals with reduced or absent NUDT15 activity may experience myelosuppression (Moriyama et al., 2016). Due to concerns regarding the efficacy and risk of toxicity, genotypes of TPMT and NUDT15 (Relling et al., 2019), and/or enzymatic functionality of TPMT, are often considered prior to initiating therapy (Kaskas et al., 2003; Relling et al., 2011). Variation in therapeutic efficacy and toxicity of thiopurine drugs is largely affected by the activity of TPMT (Lennard, 2014) and NUDT15. Yang et al. reported that of all reported cases of thiopurine drug toxicity in children with acute lymphoblastic leukemia (ALL), ∼20% can be explained by the TPMT activity and ∼22% by NUDT15 (Yang et al., 2015), while toxicity varies among ethnicities (e.g., NUDT15 polymorphism occurring more often in individuals of Asian descent).

Monitoring concentrations of the thiopurine nucleotides 6-TGN and 6-MMPN in patient samples is useful in optimizing the dose of thiopurine drugs and, therefore, balances the efficacy of thiopurine drug therapy while minimizing adverse effects. Complicating factors for therapeutic monitoring of patients on thiopurine drug therapy include a narrow therapeutic range for 6-TGN and a large interpatient variability in thiopurine drug metabolism (Gearry and Barclay, 2005). Thiopurine drugs and thiopurine nucleotides are metabolized and act intracellularly; therefore, an appropriate sample type for the measurement of thiopurine nucleotide concentrations is washed red blood cells (RBCs). RBC concentrations of thiopurine nucleotides serve as a surrogate marker of thiopurine nucleotide intracellular concentrations in nucleated cells (Balis et al., 1998; Dervieux and Boulieu, 1998; Vikingsson et al., 2013). Elevated 6-TGN concentrations may cause leukopenia and myelotoxicity, while elevated 6-MMPN may be hepatotoxic. Because of this, treatment with thiopurine drugs requires routine monitoring (Cuffari et al., 2004; Nygaard et al., 2004; Dervieux et al., 2005).

Methods for the measurement of thiopurine nucleotides in RBCs involve hydrolysis to release the purine bases (6-TG and 6-MMP) and analysis using liquid chromatography-tandem mass spectrometry (LC–MS/MS) (Shipkova et al., 2003; Dervieux et al., 2005; Neurath et al., 2005; Cangemi et al., 2012). The poor storage stability of thiopurine nucleotides in whole blood (WB) samples has been reported (de Graaf et al., 2008; Yoo et al., 2018), emphasizing the importance of pre-analytical aspects and conditions used during the sample preparation.

The goals of this study were to develop, validate, and evaluate performance of a mass spectrometry method for quantification of 6-TG and 6-MMP in RBCs. The stability of thiopurine nucleotides under various conditions was evaluated to determine the appropriate pre- and post-analytical conditions and limitations. Using historic data on the analysis of routine patient samples, we assessed the association among concentrations of thiopurine nucleotides, TPMT phenotypes, and genotypes.

## Materials and Methods

### Chemicals and Materials

The standards of 6-TG and 6-MMP, 2-amino-6-mercaptopurine-9-D-riboside hydrate (6-TGRib), 6-methylmercaptopurine riboside (6-MMPRib) and stable isotope-labeled analogs, 6-TG-^13^C_2_^15^N and 6-MMP-d_3_, were purchased from Toronto Research Chemicals, Ontario, Canada. The stock standards were prepared in dimethyl sulfoxide (DMSO) at 10 mg/ml (1 mg/ml) for the unlabeled (stable isotope-labeled) analogs. Perchloric acid (70%), dithiothreitol (DTT), and ammonium acetate were purchased from Millipore-Sigma (St. Louis, MO, United States). LC–MS grade methanol (MeOH), acetonitrile, and formic acid (FA) were purchased from Thermo Fisher Scientific (Fair Lawn, NJ, United States). Working solutions were prepared by diluting the stock solutions in an aqueous solution of 0.02 M sodium hydroxide, 7.5 mM DTT, and 20% MeOH. Working calibration standards were prepared at concentrations of 20, 50, 100, 250, 500, and 750 pmol/μL for 6-TG; and 400, 1,000, 2000, 5,000, 10,000, and 15,000 pmol/μL for 6-MMP. Quality control (QC) samples were prepared at 75, 150, and 300 pmol/μL for 6-TGRib; 1,500, 3,000, and 6,000 pmol/μL for 6-MMPRib. The working standards and the QC were prepared in pooled thiopurine nucleotide-negative RBC pools. All analyzed batches of samples contained a set of six calibration standards and three QC samples.

### Sample Preparation

Tubes with EDTA-anticoagulated WB patient samples were rocked using a rocking mixer for 15 min at room temperature (RT). One milliliter aliquots of WB samples were transferred into disposable glass tubes; RBC separation and washing were performed using the automated cell washer method as follows (Bajaj et al., 2021): Tubes with WB samples were placed in the rotor of a UltraCW^®^II instrument (Helmer Scientific, Noblesville, IN, United States) and centrifuged at 1,440 relative centrifugal force (RCF) for 4 min; during this step, RBCs were separated from plasma by centrifugal packing, followed by decantation of the plasma supernatant. The decanting step was followed by resuspension, achieved by the addition of saline into the tubes and agitation of the tubes to disperse and distribute the RBCs in the saline. Resuspension was followed by the two cycles of addition of saline, agitation, pelleting RBC, and decanting the supernatant. At the end of the last wash cycle, the tubes contained washed RBCs in a small volume of saline. The packed RBCs were diluted with 400 µL of saline, and RBCs were counted using a hematological cell counter (Ac.T Diff, Beckman Coulter, Pasadena, CA, United States). The isolated RBCs were transferred into microcentrifuge tubes.

One hundred microliter aliquots of the washed/diluted RBCs were transferred into 1.5 ml microcentrifuge tubes (Eppendorf, Enfield, CT, United States), 20 μL of internal standard was added, and the samples were vortexed and held at RT for 15 min. Then, 150 μL of 4 mM DTT and 40 μL of 70% perchloric acid were added to the samples. The tubes were vortexed for 1 min at 1,000 rpm and then centrifuged at 16,000 RCF for 5 min at RT; 100 μL of the supernatant was transferred into glass vials; the vials were capped and incubated at 100°C for 2 h (for hydrolysis) with mixing at 500 rpm using a thermo mixer (Eppendorf). During the hydrolysis, 6-MMP is chemically converted to 6-MMP-imidazole (6-MMP*) {Oliveira et al., 2004 #1465; Dervieux and Boulieu, 1998 #1469}. After cooling to RT, 140 μL of 1M ammonium acetate was added to the tubes to neutralize the acidic solution, and then 15 μL of this solution was transferred into wells of a 96-well polypropylene plate (Agilent Technologies, Santa Clara, CA, United States), and diluted with 285 μL of 0.1% formic acid in water for instrumental analysis.

### LC–MS/MS Analysis

An Agilent 6470 triple quadrupole mass spectrometer (Agilent Technologies) equipped with an electrospray (ESI) ion source was coupled to an Agilent 1260 Infinity II series HPLC system consisting of multisampler, binary pump, and column thermostat. Nitrogen was used as the drying gas, nebulizer gas, sheath gas, and collision gas; the acquisition was performed in a positive ion mode. The ion source parameters are listed in Supplementary Table S1; mass transitions with their respective fragmentor voltages and collision energies are listed in Supplementary Table S2. Chromatographic separation was performed using a XSelect Peptide HSS T3 LC column (2.5 µm, 100 Å, 2.1 × 100 mm; Waters Corporation, Milford, MA, United States). Mobile phase A was 0.1% formic acid in nanopure water (18.2 megohm ionic purity) and mobile phase B was 0.1% formic acid in acetonitrile. The flow rate was 0.4 ml/min and the column temperature was 40°C. After an initial hold at 2% B for 0.2 min, the following were performed: a gradient to 12.5% B at 3 min, column conditioning using 95% B between 3.1 and 5 min, and re-equilibration using 2% B between 5.1 and 7 min. The injection volume was 10 μL, and the total run time per sample was 7 min. The retention time of 6-TG was ∼1.6 min and that of 6-MMP was ∼2.7 min. The ratio of the peak area of the analyte to its corresponding IS was used for quantification using the MassHunter Workstation (Agilent Technologies). Calibration curves were fitted using linear regression with 1/x-weighting for 6-TG and 1/x^2^-weighting for 6-MMP*. The acceptability criterion for the ratio of mass transitions was set to ±30% (Kushnir et al., 2005).

### Method Validation

Method validation included the evaluation of precision, sensitivity, linearity, accuracy, specificity, dilution integrity, carryover, robustness, method comparison, and assessment of matrix effect. Details of the experiments on the method performance evaluated are included in the Supplementary Data. The use of residual patient samples was approved by IRB protocols (University of Utah, United States).

### Stability

The stability of thiopurine nucleotides in ETDA WB was evaluated using 15 neat patient sample pools (prepared from WB samples of patients on thiopurine drug therapy; 5–10 individual WB samples per pool); the samples were stored at 4°C for 5 h, and 4, 7, and 15 days; the observed concentrations were compared to the concentrations of 6-TG and 6-MMP in the aliquots of RBC separated from the pools at time zero, which were stored at −70°C. The stability of the thiopurine nucleotides in separated/washed RBC samples (*N* = 12) stored at −70 C was evaluated by preparing and analyzing the samples on 4 occasions over 5 months of storage and comparing the observed concentration with thiopurine nucleotide concentrations observed in the samples tested prior to the storage. The stability of the final extract (*N* = 15) was evaluated by the comparison of 6-TG and 6-MMP concentrations in fresh extracts *versus* those in extracts stored at −20°C for 2 weeks. For autosampler stability, a set of extracts (*N* = 33) was stored in a 96-well plate in an autosampler compartment at 4°C and analyzed for six consecutive days. The freeze/thaw stability of 6-TG and 6-MMP in separated/washed RBCs was evaluated in a set of patient samples (*N* = 10).

### Historical Data Analysis

Two archived datasets were retrieved retrospectively (IRB protocols approved by the University of Utah Institutional Review Board). Data in Set 1 included results from 611 specimens, submitted for routine testing for the TPMT activity (phenotype) and thiopurine nucleotide quantification (both tests ordered for same patient); and data in Set 2 included results from 64 specimens with determined TPMT genotype; thiopurine nucleotides were also measured in these patients by referral laboratory. WB samples submitted for thiopurine nucleotide testing were shipped and stored refrigerated prior to testing within 1–3 days of the collection. Both, phenotype and genotype assays were validated for the clinical use in ARUP Laboratories. Data in Set 1 consisted of 4 patient samples with low TPMT activity, 93 patients with intermediate TPMT activity, and 514 patients with normal TPMT activity. Data in Set 2 consisted of one patient with *3A/*3A, two patients with *3A/*1, three patients with *3C/*1 variant alleles, and 58 patients with no variant alleles.

## Results

### Sample Preparation, Chromatographic Separation, and LC–MS/MS Analysis

**FIGURE 1:**
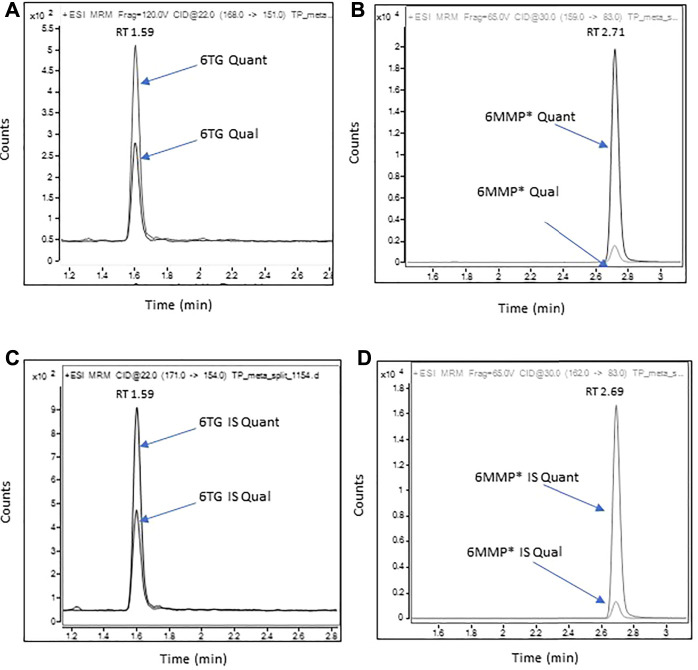
Representative chromatograms of 6-TG and 6-MMP* in a patient sample containing 107 pmol/8*10^8^ RBC of 6-TG and 6,400 pmol/8*10^8^ RBC of 6-MMP.

Representative chromatograms of 6-TG and 6-MMP* along with their corresponding IS are shown in Figure 1. The MRM transitions and the optimized instrument settings are summarized in Supplementary Table S2.

RBC separation from WB samples was performed using an automated method (Bajaj et al., 2021): the details of the method are summarized in Supplementary Figure S2. Data on the quantitative measurement of three example biomarkers (6-TGN, 6-MMPN, and Mg) with sample preparation performed using the automated vs. manual method for RBC separation and washing process demonstrate adequate performance of the automated method (Supplementary Figures S3, S4).

While developing this method, we evaluated numerous HPLC columns. Inadequate separation of the 6-TG peak from the solvent front was observed on all evaluated columns. The LC column used in the method provided the best chromatographic retention (separation from the solvent front) as compared to the other evaluated columns (Supplementary Figure S5). We found that the initial mobile phase composition had the greatest impact on the retention time of 6-TG and 6-MMP*, the 6-MMP* peak width, and the signal-to-noise ratio for the 6-TG peak, while the end-of-gradient mobile phase composition had a significant effect on the retention time of the 6-MMP* peak.

### Assay Validation

Summary data for the evaluation of the method’s imprecision are included in Supplementary Table S5; imprecision at all evaluated concentrations for 6-TG and 6-MMP was ≤3%. The limit of quantitation (LOQ) for 6-TG and 6-MMP was 20 and 400 pmol per 100 μL (0.2 and 4 μmol/L), respectively, of RBC lysate; data on imprecision and accuracy at the LOQ are summarized in Supplementary Table S6; the signal-to-noise ratio at the LOQ for the transitions of both analytes was ≥10. Data on method linearity are summarized in Supplementary Figure S6. Linear regression equations and coefficients of determination for correlation between the expected and the observed concentrations were y = 1.01x + 3.28, *R* ^2^ = 0.996 and y = 0.97x + 316, *R* ^2^ = 0.998, for 6-TG and 6-MMP, respectively.

The method’s accuracy was evaluated by analysis of individual thiopurine nucleotide-negative patient RBC lysates spiked at six different concentrations with 6-TGRib and 6-MMPRib conjugates. The samples were prepared and analyzed in triplicate, and the expected and observed concentrations agreed within 1 and 15%, for 6-TG and 6-MMP, respectively, with imprecision among the replicates of <5% (Supplementary Figure S7).

Potential for interference was evaluated in the WB samples of individuals not on thiopurine drugs therapy (*n* = 110), and in lysed RBC pools spiked with common drugs and drug metabolites (Supplementary Table S7), there were no peaks in the mass transitions within the acquisition time of the assay.

Dilution integrity was evaluated by the analysis of five individual neat patient RBC lysate samples containing an elevated concentration of 6-TGN and 6-MMPN using 5-fold and 10-fold dilution, respectively, with a lysed thiopurine nucleotide-negative RBC pool. After normalizing the observed concentrations for the dilution factor, the agreement for both 6-TG and 6-MMP was within 17% (Supplementary Table S8 *).*

No carryover to the following sample was observed after injection of samples containing 5,000 pmol/100 μL of 6-TG and 100,000 pmol/100 μL of 6-MMP.

**FIGURE 2:**
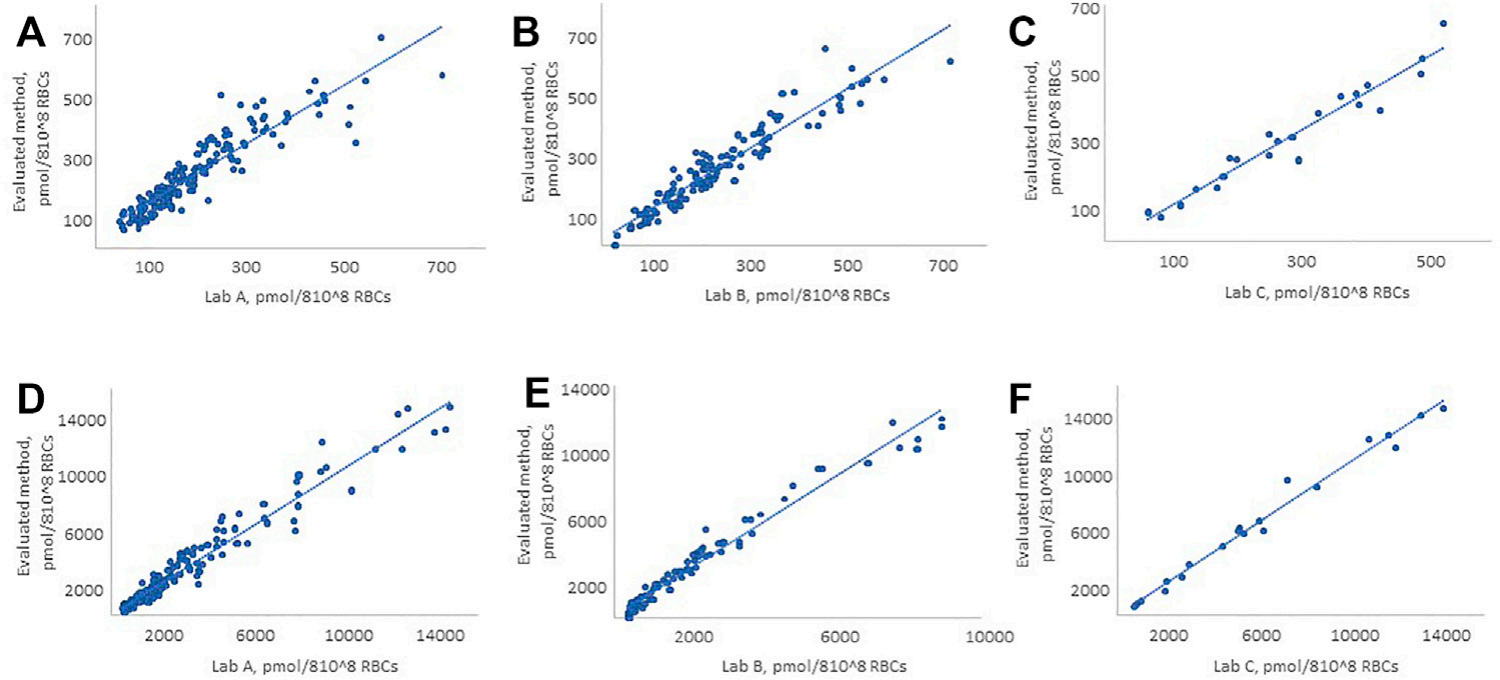
Comparison of the evaluated method with LC–MS/MS methods of Lab A, Lab B, and Lab C for 6-TG and 6-MMP. **(A, D)** 6-TG and 6-MMP comparison with Lab A, **(B, E)** 6-TG and 6-MMP comparison with Lab B, and **(C, F)** 6-TG and 6-MMP comparison with Lab C. The figure shows only the patient samples where the observed concentrations were within the analytical measurement range of the method.

The method comparison was performed using residual de-identified WB samples from patients receiving thiopurine drug therapy. The evaluated method was compared to LC–MS/MS methods of three commercial laboratories, which perform the test routinely (WB samples were split and sent refrigerated to the external laboratories). For comparison purposes, these laboratories are referred to as Lab A, Lab B, and Lab C. The developed method showed good agreement with Lab A and C, for both 6-TG and 6-MMP, and with Lab B for 6-TG, while concentrations of 6-MMP were underestimated using the method of Lab B (Figure 2). The number of patient samples tested, Deming regression equation slopes, intercepts, correlation coefficients (R), and percent bias are summarized in Table 1.

**TABLE 1:** Summary of the results of method comparison.

Specifics	Lab A		Lab B		Lab C	
	6-TG	6-MMP	6-TG	6-MMP	6-TG	6-MMP
Number of samples	173	154	123	83	22	21
Slope	1.06	1.04	1.02	1.40	1.14	1.06
Intercept	26.6	6.7	9.9	112	−20.5	−55.4
(R)	0.914	0.975	0.950	0.985	0.971	0.992
% Bias	19.2	4.5	6.6	45.8	7.4	5.2

### Samples’ Stability

We assessed WB samples’ stability by evaluating the measured 6-TG and 6-MMP concentrations in WB sample pools (*n* = 15) stored refrigerated (4°C) for 5 h, and 4, 7, and 14 days. After 7 (14) days of storage at 4°C, the measured concentrations of 6-TG (6-MMP) decreased by 0.5 (11)% and 3 (13)%, respectively (Supplementary Figure S8).

We also evaluated the analyte stability in washed RBC lysates stored at −70°C for up to 150 days. After 150 days of storage, the observed 6-TG and 6-MMP concentrations decreased by <10%, as compared to the Day 1 (Supplementary Figure S9).

In an experiment, on evaluation of 6-TGN and 6-MMPN stability in individual prepared samples stored at −20°C (*n* = 15) for 15 days, we observed <10% reduction in concentrations of 6-TG and 6-MMP (Supplementary Figure S10). The evaluation of 6-TG and 6-MMP stability in the samples prepared for analysis, stored at 4°C for up to 120 h, demonstrated no change in the measured 6-TG and 6-MMP concentrations (Supplementary Figure S11). The evaluation of the freeze-thaw stability (3 cycles) of 6-TGN and 6-MMPN in RBCs demonstrated ∼8% (∼6%) reduction in the concentration of 6-TG (6-MMP) (Supplementary Figure S12).

### TPMT Phenotype/Activity and Thiopurine Metabolite Concentrations

Retrospective data analysis was performed to assess the correlation between the results of TPMT activity testing and thiopurine metabolite concentrations for the first occurrence of therapeutic drug monitoring. Of note, the dataset does not include serial patient monitoring. The dataset contained results corresponding to patient samples that were tested for both TPMT phenotype and thiopurine nucleotides (during the thiopurine drug therapy, *n* = 611). Out of the entire dataset, 514 patients had shown the TPMT enzyme activity within the therapeutic range (24–44.0 U/mL), 93 had a reduced activity (17.0–23.9 U/mL) and 4 had a low TPMT activity (<17.0 U/mL). There were no patients in the dataset who had a high TPMT activity (>44.0 U/mL).

Out of the 514 patients with normal TPMT activity, 90 patients (17.5%) had 6-TG concentrations within the therapeutic range (235–450 pmol/8 × 10^8^ RBCs), 396 patients (77.0%) had subtherapeutic 6-TG concentrations (<235 pmol/8 × 10^8^ RBCs), and 28 patients (5.4%) had supratherapeutic 6-TG concentrations (>450 pmol/8 × 10^8^ RBCs). Among the patients with intermediate TPMT activity, 23.7% had 6-TG concentrations within the therapeutic range, 60.2% were subtherapeutic, and 16.1% had 6-TG concentrations >450 pmol/8 × 10^8^ RBCs. Among the four patients with low TPMT activity, two had 6-TG concentrations within the therapeutic range and two had subtherapeutic concentrations.

The distribution of 6-MMP concentrations demonstrated that among 514 patients with normal TPMT activity, 4.1% had concentrations >5,700 pmol/8 × 10^8^ RBCs. Among 93 patients with intermediate TPMT activity, 3.2% had 6-MMP concentration >5,700 pmol/8 × 10^8^ RBCs. All four patients with low TPMT activity had 6-MMP concentrations <5,700 pmol/8 × 10^8^ RBCs.

**FIGURE 3:**
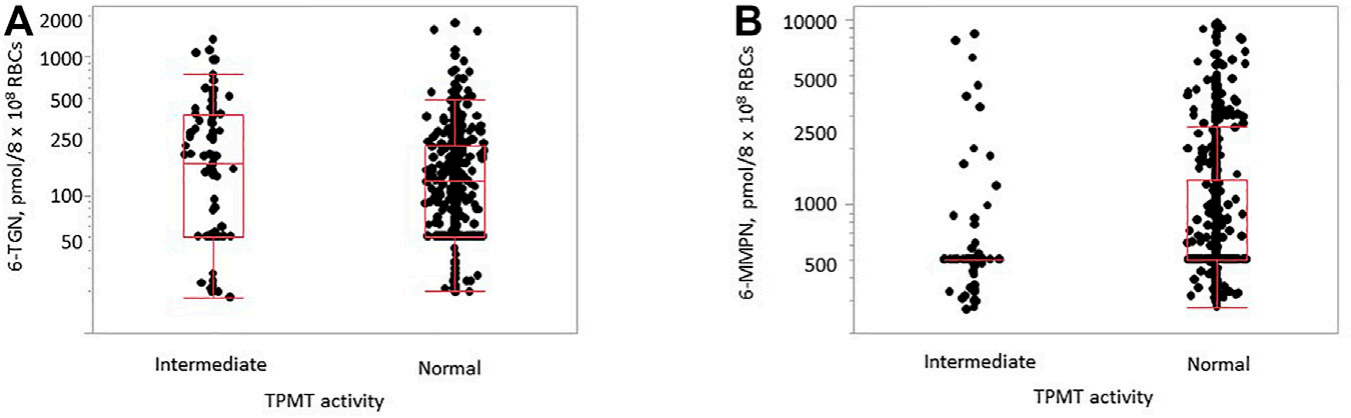
Distribution of 6-TG [**(A)**, *p* = 0.096] and 6-MMP [**(B)**, *p* < 0.0001] concentrations in samples with normal (*n* = 514) and intermediate (*n* = 93) TPMT activity in samples of children and adults.

The median (interquartile range) for the distribution of 6-TG and 6-MMP concentrations was 126 (50–238) pmol/8 × 10^8^ RBCs and 500 (500–1,160) pmol/8 × 10^8^ RBCs, respectively. The difference in the observed 6-TG concentrations between TPMT groups with normal and intermediate activity was not statistically significant (*p* = 0.096), while it was statistically significant for 6-MMP (*p* < 0.0001, Figure 3). There was no significant difference in the TPMT activity between males and females (*p* = 0.824 for 6-TG and *p* = 0.734 for 6-MMP). A statistically significant difference in 6-MMP concentrations was observed between samples of children and adults with normal TPMT activity (*p* = 0.015, Supplementary Figure S13), with higher concentrations observed in pediatric samples; no statistically significant difference was observed in 6-TG concentrations (*p* = 0.147).

In the evaluated dataset, there were 64 patients on thiopurine drug therapy who had thiopurine metabolite measurements performed along with the test for the TPMT genotype. Out of 58 patient samples of this set, who were classified as TPMT *1/*1 (wildtype), five patient samples (8.6%) had 6-TG concentrations within the therapeutic range (235–450 pmol/8 × 10^8^ RBCs). Forty-four patients (75.9%) had subtherapeutic 6-TG concentrations (<235 pmol/8 × 10^8^ RBCs) and nine patients (15.5%) had 6-TG concentrations >450 pmol/8 × 10^8^ RBCs. When evaluating 6-MMP concentration, 45 patients (77.6%) had 6-MMP concentration <5,700 pmol/8 × 10^8^ RBCs and 13 patients (22.4%) had >5,700 pmol/8 × 10^8^ RBCs. Out of the patients included in this dataset, 91.1% had 6-TG concentrations outside of the therapeutic range; the median (interquartile range) for 6-TG and 6-MMP concentrations were 72 (50–253) pmol/8 × 10^8^ RBCs, and 500 (500–3,218) pmol/8 × 10^8^ RBCs, respectively.

## Discussion

Therapeutic drug monitoring for thiopurine metabolites is important to guide therapy to manage myelosuppression and to minimize the risk of hepatotoxicity {Relling et al., 2019 #8}. The novel aspects of this study include the following: 1) development of a LC–MS/MS method for the measurement of thiopurine nucleotides that use an automated method for separation and washing of RBCs from WB samples; 2) optimization of chromatographic separation, which enhanced the analytical sensitivity and reduced the potential for ion suppression; 3) optimization of the conditions for thiopurine metabolite conjugate hydrolysis and 6-MMPN conversion to 6-MMP*; 4) assessment of 6-TGN and 6-MMPN stability in WB samples, washed RBCs, and in the samples prepared for the analysis; and 5) assessment of association between the TPMT phenotype and genotype and thiopurine nucleotide concentrations in routine patient samples.

The developed method utilizes automated RBC separation and washing from WB samples {Bajaj et al., 2021 #22}. Processing time for 24 WB samples is ∼15 min, as compared to 2–2.5 h, required for manual sample processing (O'Connell et al., 1965; Thompson et al., 1984; Bosch et al., 1992; Connor et al., 1994). Our data suggest that this automated RBC separation and washing method holds a great promise for reducing the labor required for the sample processing in methods using RBC separation and washing.

We observed that performing RBC washes during the RBC separation was important for the adequate quantitative performance of the assay, as plasma may contain thiopurine metabolites, which could cause falsely elevated thiopurine nucleotide concentrations. In our method, there was no significant difference in the thiopurine nucleotide concentration between RBCs separated from samples that were washed two and three times; therefore, the method uses two washes.

In samples from patients on thiopurine drug therapy, 6-MMP concentrations in RBC samples were 10–20 times greater than 6-TG, which makes the simultaneous analysis of 6-TG and 6-MMP challenging. To address this issue, we evaluated 6-MMP mass transitions based on the first isotope (M+1) of 6-MMP* instead of mass transitions based on monoisotopic ions, which are typically used in MS/MS methods for quantitative analysis. The observed M+1 peak abundances were ∼10 times lower than those from monoisotopic ions, resulting in comparable peak intensities of 6-TG and 6-MMP* in the calibrators, QC, and patient samples.

Our data demonstrated that the adequate recovery of 6-TG and 6-MMP from the respective conjugated forms of the thiopurine nucleotides present in RBC, and quantitative 6-MMPN conversion to 6-MMP*, requires hydrolysis at 100°C for 2 h, while hydrolysis at lower temperatures or shorter times resulted in incomplete hydrolysis, incomplete conversion to 6-MMP*, and underestimation of 6-TG and 6-MMP concentrations.

Numerous methods for the analysis of thiopurine metabolites in RBCs have been published (Erdmann et al., 1990; Lavi and Holcenberg, 1985; Lennard and Singleton, 1992; Moon et al., 2019; Thomas et al., 2018). Dervieux et al. developed the first LC–MS/MS method for the analysis of thiopurine nucleotides in RBCs and demonstrated its clinical utility (Dervieux et al., 2005). Kirchherr et al. (2013) changed the sample type from washed RBCs to WB with subsequent normalization to account for the WB sample variation in the RBC content. In our experience, measurement of thiopurine nucleotides in WB leads to overestimation of the concentrations in some samples. Hofmann et al. (2012) developed an LC–MS/MS method for simultaneous quantification of eleven thiopurine drug metabolites, including monophosphate, diphosphate, and triphosphate nucleotides of 6-TG and 6-MMP, and evaluated their clinical significance. Additional studies are needed to assess the clinical utility of measuring the individual phosphorylated nucleotide conjugates. Methods for the measurement of thiopurine metabolites in plasma have been developed (Tsutsumi et al., 1982; Sorouraddin et al., 2011; Al-Ghobashy et al., 2016), while very few plasma patient samples were analyzed as part of these studies, and clinical utility of these measurements was not demonstrated.

As reported by Simsek et al., poor agreement among methods of different laboratories may be attributed to the sample stability, standardization, or poor control of the hydrolysis of 6-TGN and 6-MMPN conjugates (Simsek et al., 2017). We observed systematic bias in 6-MMP concentrations in the method compared with one of the external laboratories. Due to insufficient method information, we cannot comment on the specific cause of this disagreement. Based on our data, despite a lack of certified reference materials and proficiency testing programs, the among-laboratory agreement in 6-TG and 6-MMP concentrations was strong.

The poor stability of thiopurine nucleotides in WB samples was reported in several publications (Pike et al., 2001; Hofmann et al., 2012; Yoo et al., 2018). Pike et al. (2001) reported a decrease in 6-TGN concentration by 2–4% per day in WB stored at ambient temperature, and Yoo et al. (2018) recommended that RBCs should be separated within four days of the blood draw, if WB samples are stored refrigerated. Our data are in agreement with those of Yoo et al., suggesting the adequate sample stability with storage at 4 C for up to 4 days after receipt by the laboratory.

Pike et al. (2001) reported that 6-TGN concentration decreased by about 12% after six months of storage at −80°C and that 6-MMPN concentration did not change after six months. Yoo et al. reported a decrease in 6-TGN (6-MMPN) concentrations by 5 (10)% after storage at −70°C for 180 days (Yoo et al., 2018). Our results on the stability of thiopurine nucleotides in separated/washed RBC samples are in agreement with the data from previous publications.

Because thiopurine drug therapy may cause life-threatening myelosuppression and hepatotoxicity, the assessment of TPMT enzyme activity prior to the initiation of therapy is recommended to identify patients at risk. Patients with low TPMT activity are expected to be at risk of bone marrow toxicity and are recommended to avoid the use of thiopurine drug therapy or to significantly reduce the dose. Patients with intermediate TPMT activity could also be at risk of bone marrow toxicity and are recommended to reduce the dose and have frequent thiopurine metabolites monitoring. Patients with high TPMT activity are not considered to be at a risk of bone marrow toxicity and standard dosing, and periodic thiopurine metabolite monitoring is recommended (Lee et al., 2021; Franca et al., 2021).

Data from our retrospective analysis demonstrated that TPMT activity better explained the concentrations of 6-MMP than concentrations of 6-TG. In agreement with earlier publications, there was not a significant difference in the TPMT activity between males and females (Wiwattanakul et al., 2017; Wu et al., 2019). No statistically significant difference in 6-TG concentrations was observed between children and adults in samples with normal or intermediate TPMT activity; however, statistically significantly higher 6-MMP concentrations were observed in pediatric samples than in samples of adults. Due to limited patient information in our laboratory information system, we cannot determine the exact reason for this observation. This observation could be related to the difference in the dosage and treatment regimens in children with ALL, as compared to conditions for which thiopurine drug therapy is used in adults.

In addition to the TPMT phenotype, TPMT and NUDT15 genotypes have been used to identify patients at risk for adverse effects before initiation of thiopurine drug therapy (Wiwattanakul et al., 2017). Dervieux et al. observed association between the TPMT genotype and metabolite concentrations, illustrating the utility of pharmacogenetics in the management of patients for whom thiopurine drug treatment was recommended (Dervieux et al., 2005). We did not observe statistically significant differences in 6-TG and 6-MMP concentrations among the groups with different genotypes, which is likely explained by the relatively small size of our dataset.

Surprisingly, 6-TG concentration in 81.3% of the tested samples was outside of the therapeutic range, which highlights the clinical need for routine monitoring of thiopurine metabolites to help optimize therapy and to achieve the adequate clinical outcomes. Alternatively, the large percentage of samples with 6-TG concentration below the therapeutic range could be related to 6-TGN degradation during the time between the blood draw and analysis of the samples at a clinical laboratory. Future studies are needed, using samples from patients on thiopurine drug therapy, for whom clinical information is available, to determine the cause of the high frequency of observed 6-TGN concentrations below the therapeutic range.

Our study had some limitations. Due to the unavailability of large volumes of neat WB samples from patients on thiopurine drug therapy, evaluation of the method accuracy, precision, sensitivity, and linearity was performed using thiopurine nucleotide-negative RBC lysate samples spiked with 6-TG and 6-MMP ribose conjugates. The use of ribose conjugates allowed us to assess the effect of hydrolysis on the method’s performance in all validation experiments.

In the data on retrospective data analysis, we did not have access to the clinical information of the participants [disease, dosing, dosing frequency, treatment compliance, and recent red blood cell transfusion (since blood cell transfusions can reflect the TPMT activity of the donor instead of the recipient)]. Moreover, it is unknown whether these patients were taking other medications or underwent treatments, which could alter the TPMT activity (Lewis et al., 1997; Lennard et al., 2001; Brouwer et al., 2005).

## Conclusion

We developed and validated a LC–MS/MS method for quantification of 6-TG and 6-MMP in RBCs. As part of this method, we developed an automated procedure for RBC separation from WB samples and washing of the separated RBCs. Our data suggest that this automated method for RBC separation and washing has an adequate performance, and significantly reduces labor and time as compared to the manual methods. We observed reasonable inter-laboratory agreement in the measured concentrations of 6-TG and 6-MMP, while the among-laboratory agreement could likely be improved through standardization and harmonization. Some of the contributing factors impacting the accurate quantification of thiopurine nucleotides are a lack of certified reference materials and proficiency testing programs, poor stability of WB patient samples and the analyte standards, and among-laboratory differences in the methodologies. A review of the retrospective data revealed higher 6-MMP concentrations in samples of children than in samples of adults, and a high frequency of samples with 6-TG concentrations below the therapeutic range; future studies are needed to determine the cause of the aforementioned observations.

## Data Availability

The original contributions presented in the study are included in the article/Supplementary Material; further inquiries can be directed to the corresponding authors.
