# Supplementary material for: LC–MS/MS Method for Measurement of Thiopurine Nucleotides (TN) in Erythrocytes and Association of TN Concentrations With TPMT Enzyme Activity
Source: Front Pharmacol. 2022 Mar 21;13:836812. doi: 10.3389/fphar.2022.836812 (PMC8978547; doi:10.3389/fphar.2022.836812)
Supplement: Supplementary file 1 [file DataSheet1.docx]

**LC-MS/MS method for measurement of Thiopurine Nucleotides (TN) in erythrocytes and association of TN concentrations with TPMT enzyme activity**

Amol O. Bajaj^a*^, Mark M. Kushnir^a,b^, Erik Kish-Trier^a^, Rachel N. Law^a^, Lauren M. Zuromski^a^, Alejandro R. Molinelli^c^, Gwendolyn A. McMillin^a,b^, Kamisha L. Johnson-Davis^a,b*^

^a^ARUP Institute for Clinical and Experimental Pathology, Salt Lake City, Utah, USA.

^b^ University of Utah Health Sciences Center, Department of Pathology, Salt Lake City, Utah, USA.

^c^ Department of Pharmaceutical Sciences, St. Jude Children’s Research Hospital, Memphis, TN.

***Correspondence:** Kamisha L. Johnson-Davis, [kamisha.johnson-davis@aruplab.com](mailto:kamisha.johnson-davis@aruplab.com) and Amol O. Bajaj, [amol.bajaj@aruplab.com](mailto:amol.bajaj@aruplab.com), ARUP Institute for Clinical and Experimental Pathology, 500 Chipeta Way, Salt Lake City, Utah-84108, USA*.*

**Table of Content**

**Supplementary Method Validation.** List of validation parameters tested.

**Supplementary Figure S1.** Metabolism of thiopurine drugs (metabolites highlighted in the pathway are measured in the assay). Abbreviations: 6-TG, 6-Thioguanine; 6-MMP, 6-Methylmercaptopurine; MP, 6-mercaptopurine; AZA, Azathioprine; TPMT, Thiopurine S-methytransferase; 6-MeTIMP, 6-methylthioinosine monophosphate; 6-MeTITP, 6-methylthioinosine triphosphate; 6-TXMP, 6-thioxanthosine monophosphate; 6-TITP, 6-thioinosine triphosphate; 6-TIMP, 6-thioinosine monophosphate; HPRT- hypoxanthine phosphoribosyl transferase, XO – xanthine oxidase, GMPS, guanosine monophosphate synthetase; IMPDH, inosine monophosphate dehydrogenase.

**Supplementary Figure S2.** Schematic representation of the automated method for separating and washing RBC from WB samples.

**Supplementary Figure S3.** Magnesium (Mg) concentrations in RBCs separated from WB samples using manual and automated RBC separation and washing.

**Supplementary Figure S4.** Concentration (pmol/µL) of 6-TG (A) and 6-MMP* (B) in the samples tested for evaluation of the potential for cross-contamination in the process of automated RBC separation and washing.

**Supplementary Figure S5**. Chromatograms of 6-TG and 6-MMP* using F5 (Phenomenex, 2.1X100 mm, 2.5 µm) column and HSST3 column (Waters, 2.1X100 mm, 2.6 µm).

**Supplementary Figure S6.** Simple Linearity Regression for AMR n = 3 for 2 days for (A) 6-TG and (B) 6-MMP*.

**Supplementary Figure S7.** Expected vs observed concentrations of 6-TG (A) and 6-MMP* (B) in samples analyzed for evaluation of accuracy of the method.

**Supplementary Figure S8.** 6-TG and 6-MMP stability in WB samples: change in 6-TG (A) and 6-MMP (B) concentrations in WB samples stored at 4°C.

**Supplementary Figure S9.** 6-TG and 6-MMP stability in washed RBC samples: change in concentration of 6-TG and 6-MMP in processed samples stored at -70°C for 5 months (as compared to Day 1).

**Supplementary Figure S10.** 6-TG and 6-MMP stability in prepared samples: change in concentration of 6-TG and 6-MMP in extracted samples stored at -20°C for two weeks (as compared to Day 1).

**Supplementary Figure S11.** 6-TG and 6-MMP stability in the final prepared samples: 6-TG (A) and 6-MMP* (B) concentration at T 120h as compared to T 0h when kept standing in autosampler at 4°C.

**Supplementary Figure S12.** Effect of freeze-thaw cycles on 6-TG and 6-MMP stability in washed RBC samples: 6-TG (A) and 6-MMP* (B) concentration after three freeze-thaw.

**Supplementary Figure S13.** Distribution of 6-TG (A), and 6-MMP* (B) concentrations in patient samples by age group, in patient samples with normal [6-TG (p=0.175), 6-MMP* (p=0.015)] and intermediate [6-TG (p=0.956), 6-MMP* (p=0.923)] TPMT activity.

**Supplementary Table S1.** Source parameters used in the method.

**Supplementary Table S2.** Mass transitions and instrument settings used in the method.

**Supplementary Table S3.** Effect of the decanting centrifugal force on RBCs recovery.

**Supplementary Table S4.** Effect of the centrifugation time on RBCs recovery.

**Supplementary Table S5.** The within-run, between-run and total %CV for analysis of RBC samples containing low and high concentrations of 6-TGRib and 6-MMPRib, and pool of WB samples from patients on thiopurine drug therapy.

**Supplementary Table S6.** The within-run %CV, between-run %CV, Total %CV and Avg. % Accuracy of Cal 1 for Multi-day sensitivity (samples analyzed in 5 replicates on 4 days) for 6-TG and 6-MMP*.

**Supplementary Table S7.** Common drugs evaluated for potential interference with the assay.

**Supplementary Table S8.** Evaluation of the sample’s dilution integrity.

*Method Validation*

*Precision*

Within-run, between-run and total imprecision of the assay was evaluated by analysis of RBC samples pool spiked at three different concentrations [low ~50 (~2000) pmol/100 µL, medium ~100 (6000) pmol/100 µL and high ~500 (~12,000) pmol/100 µL] of 6-TG (6-MMP), respectively. The samples were prepared and analyzed in five replicates over four days. The within-run, between-run and total imprecision was calculated. Acceptability criteria for the imprecision was ≤ 15%.

*Sensitivity*

The method sensitivity was evaluated by analysis of a pool of RBC samples spiked with 6-TG and 6-MMP to concentrations of 20 pmol/100 µL and 400 pmol/100 µL, respectively. The samples were analyzed in five replicates over four days as well as analyzed in 10 replicates in one day. Acceptability criteria for the imprecision was ≤ 20% and for accuracy ≤ ± 20% of the expected concentration.

*Linearity*

The method linearity was evaluated by analysis of a pool of RBC samples spiked with 6-TG and 6-MMP to concentrations of 20-750 pmol/100 µL and 400-15,000 pmol/100 µL, respectively. The samples were analyzed in three replicates over two days. Acceptability criteria for the imprecision was ≤ 15% (LLOQ ≤ 20%) and for accuracy ≤ ± 15% (LLOQ ≤ 20%) of the expected concentration.

*Accuracy*

The method accuracy was evaluated by analysis of individual negative patient RBC samples spiked with 6-TGRib and 6-MMPRib to concentrations of 20-750 pmol/100 µL and 400-15,000 pmol/100 µL, respectively (6 concentration levels in 4 patients each). The samples were analyzed in single replicates over two days. Acceptability criteria for slope was 0.8–1.2, with a y-intercept that was less than or equal to 3 X LLOQ for each analyte and a correlation coefficient value greater than or equal to 0.95.

*Specificity*

The specificity of the analytical method is its ability to differentiate between the analyte and the other substances in the sample matrix and thereby generate signals which are free from interference. Evaluation of specificity was performed by spiking potential interfering compounds in lysed RBCs post washing and prior to hydrolysis. Also, negative patient whole blood samples were processed through the entire extraction process including washing and evaluated for presence of any interference. The analyte response in the spikes and negative patient samples was calculated for each analyte with an acceptability cutoff of less than or equal to ± 20% of LLOQ area.

*Dilution*

A specimen dilution protocol was evaluated by taking a high concentration for each of the two analytes, then the specimens were mixed with lysed RBCs used as negative control to make a 5-fold and 10-fold dilution. Each dilution, along with the undiluted sample, was analyzed three times in a single batch. The calculated target concentration for each dilution level was compared to the mean of the observed concentration, then the percent deviation was determined. The acceptance criteria were set at less than or equal to ± 15% deviation.

*Carryover*

Carryover was determined by analyzing two pools of lysed RBCs used as negative control, one spiked approximately to five times the concentration of the highest calibrator (H) and one spiked to level of second calibrator (L). These pools were run in the pattern of L1, L2, L3, H1, H2, L4, H3, H4, L5, L6, L7, L8, H5, H6, L9, H7, H8, L10, H9, H10, L11. If carryover was present, the low samples, that were immediately following the high samples (L4, L5, L9, L10, L11) would quantify at a higher concentration than the low samples following the low samples (L2, L3, L6, L7, L8) in the sequence. The carryover evaluation is said to pass if the observed carryover is less than 3X low-low SD.

*Chromatographic separation*

The robustness of the method determines the susceptibility of a method to small changes such as pH values, temperature, mobile phase composition, etc. which might occur during routine analysis at the laboratory. In the present study, robustness was investigated by deliberately making the following alterations in the analytical method: (i) Initial %A: ± 2%, (ii) End gradient %A: ± 2% (iii) Column Temperature: ± 3 °C At each condition, the system suitability standard solution of containing both the analytes was administered into the chromatographic system in duplicates. The robustness of the method was assessed by calculating the % RSD of the peak area after three consecutive injections of the standard solution in case of (i) and (ii) and shift in RT in case of (iii).

*Method Comparison*

As there is no reference method available, results of our method were compared with those of other institutions using whole blood specimens collected from patients and/or lysed RBCs stored at -70 °C. Use of deidentified residual patient samples was approved by IRB of University of Utah; concentrations of 6-TG and 6-MMP were not available at the time of the sample’s analysis.

*Matrix Effect*

The matrix effect was determined by post-column infusion of analyte at concentration that produces response equivalent to middle of calibration curve. 6-TG was used for this purpose. Whole blood patient samples not containing the analytes of interest was used and extracted according to the sample preparation method and injected while standard of 6-TG was infused in the ion source of the instrument.

*Statistical Analysis*

Analyte concentrations we calculated using Microsoft Excel (Microsoft, WA, USA) and Agilent Quantitative Analysis Software. The between-groups comparison was performed using Wilcoxon test; p-values <0.05 were considered as representation of statistical significance.


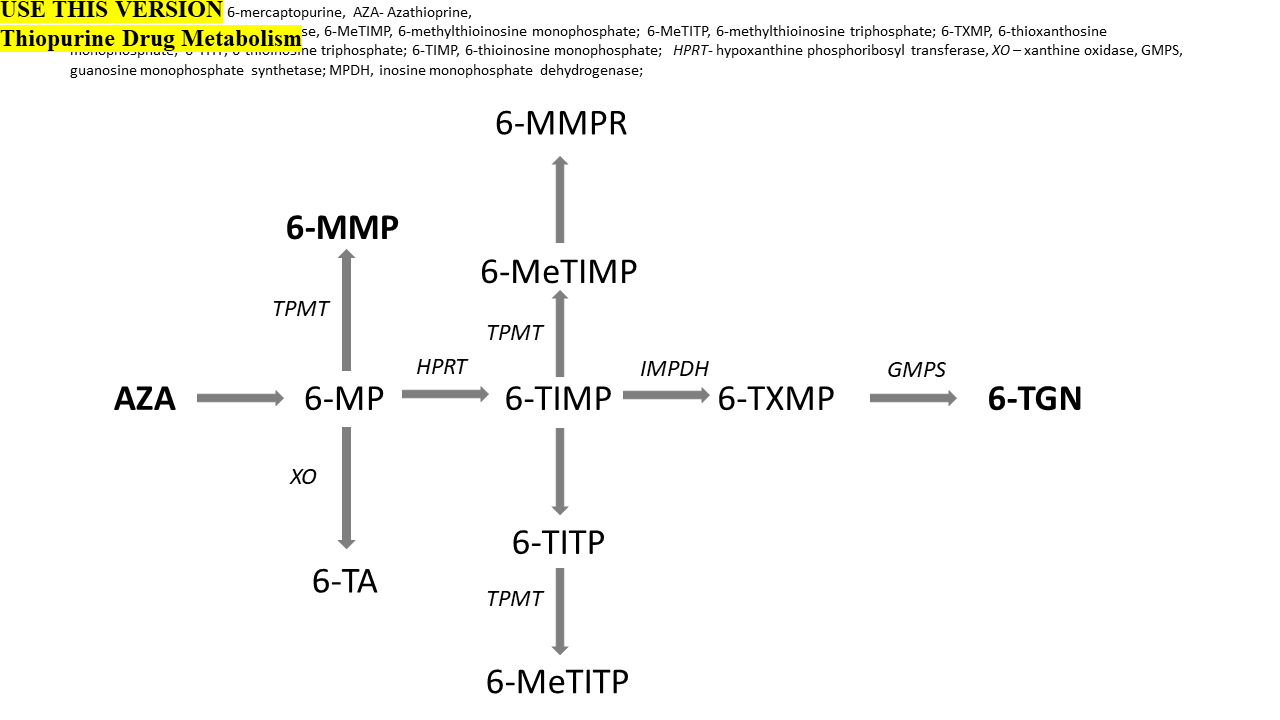


*Supplementary Figure S1. Metabolism of thiopurine drugs. Abbreviations: 6-TGN, 6-Thioguanine nucleotides; 6-MMP, 6-Methylmercaptopurine; MP, 6-mercaptopurine; AZA, Azathioprine; TPMT, Thiopurine S-methytransferase; 6-TA, 6-thiouric acid; 6-MeTIMP, 6-methylthioinosine monophosphate; 6-MeTITP, 6-methylthioinosine triphosphate; 6-TXMP, 6-thioxanthosine monophosphate; 6-TITP, 6-thioinosine triphosphate; 6-TIMP, 6-thioinosine monophosphate; HPRT- hypoxanthine phosphoribosyl transferase, XO – xanthine oxidase, GMPS, guanosine monophosphate synthetase; IMPDH, inosine monophosphate dehydrogenase*

*Supplementary Figure S2. Schematic representation of the automated method for separating and washing RBC from WB samples.*

*Supplementary Figure S3. Magnesium (Mg) concentrations in RBCs separated from WB samples using manual and automated RBC separation and washing.*

*
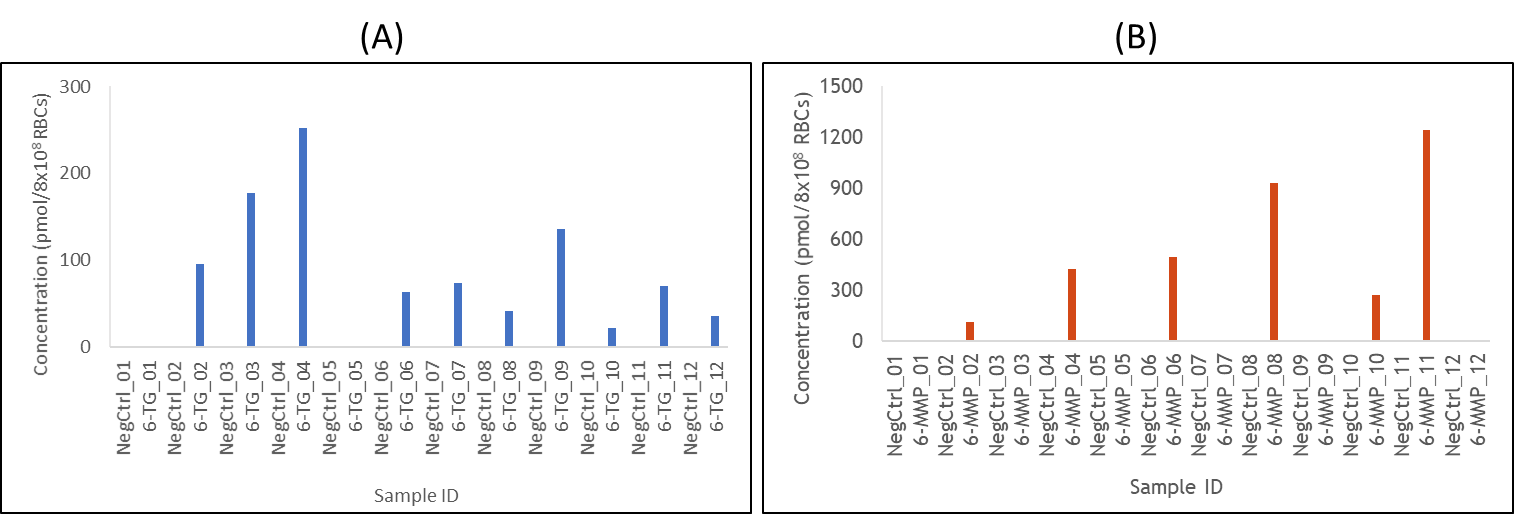
*

*Supplementary Figure S4. Concentration (pmol/8x10^8^ RBCs) of 6-TG (A) and 6-MMP* (B) in the samples tested for evaluation of the potential for cross-contamination in the process of automated RBC separation and washing.*

*
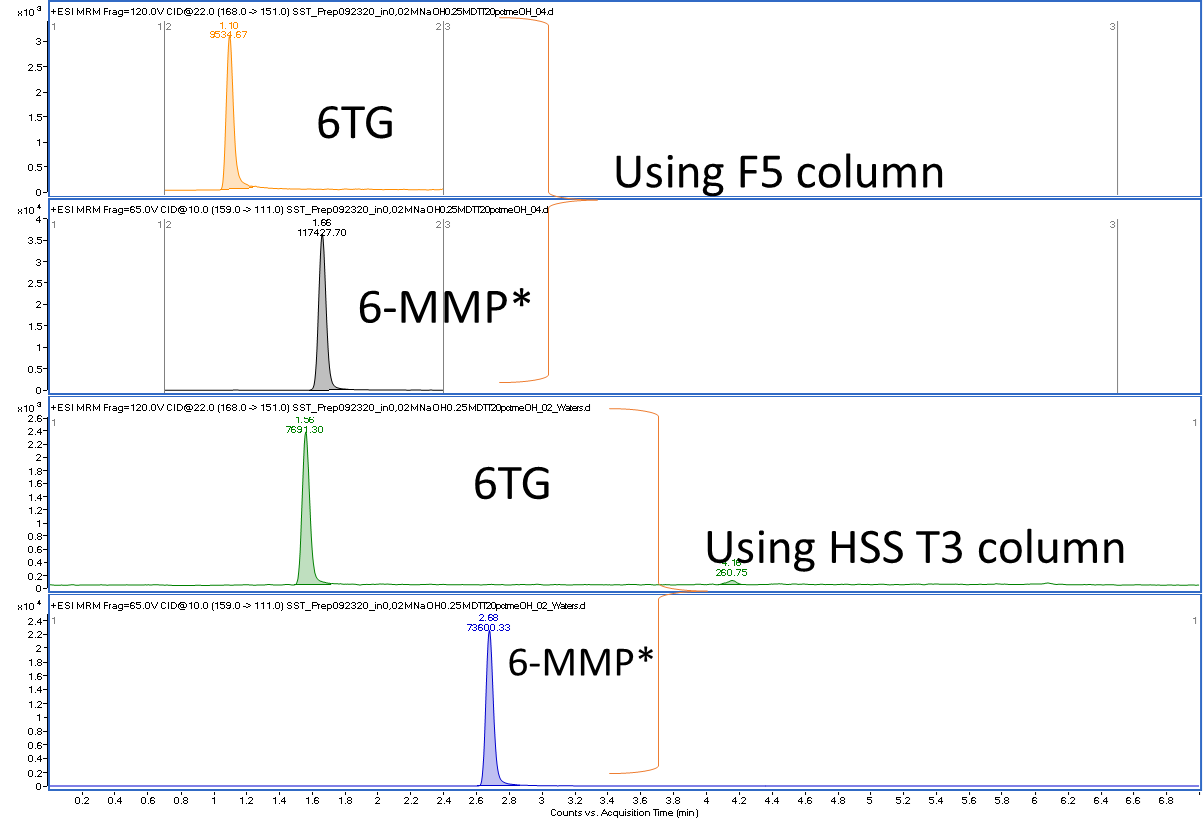
*

*Supplementary Figure S5. Chromatograms of* *6-TG and 6-MMP* using F5 (Phenomenex, 2.1X100 mm, 2.5 µm) column and HSST3 column (Waters, 2.1X100 mm, 2.6 µm) with same gradient and mobile phase composition.*


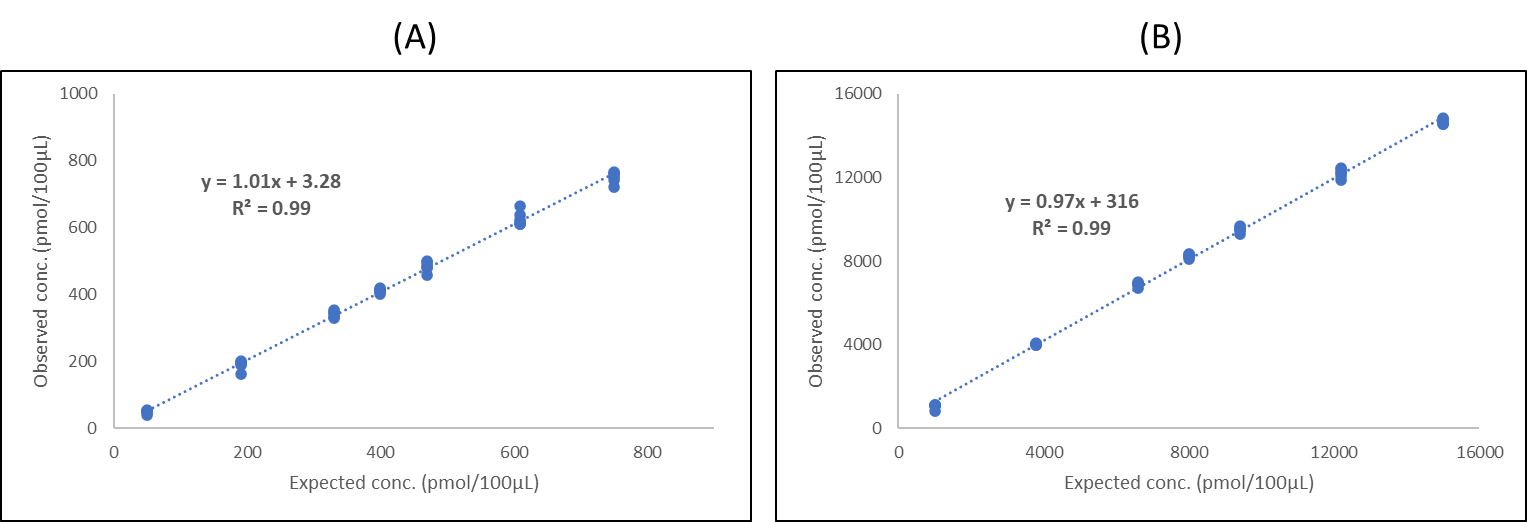


*Supplementary Figure S6. Evaluation of the method linearity (samples analyzed in 3 replicates on 2 days) (A) 6-TG and (B) 6-MMP**

*
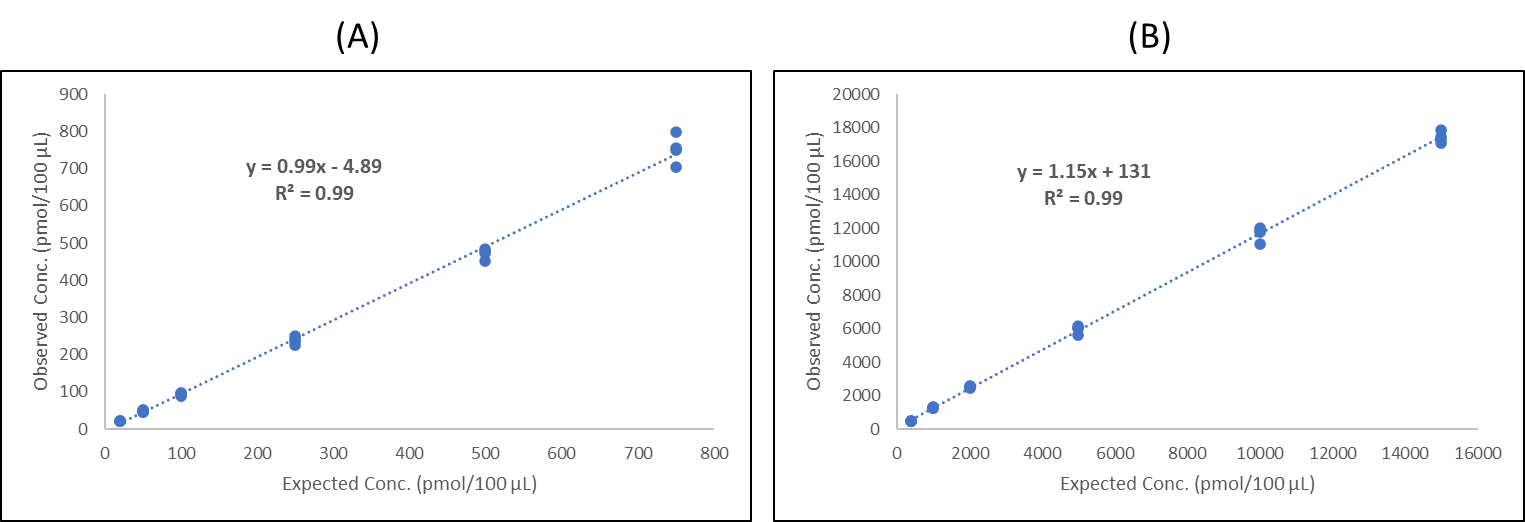
*

*Supplementary Figure S7. Expected vs observed concentrations of 6-TG (A) and 6-MMP* (B) in samples (samples analyzed in 4 replicates on 2 days) analyzed for evaluation of accuracy of the method.*

*
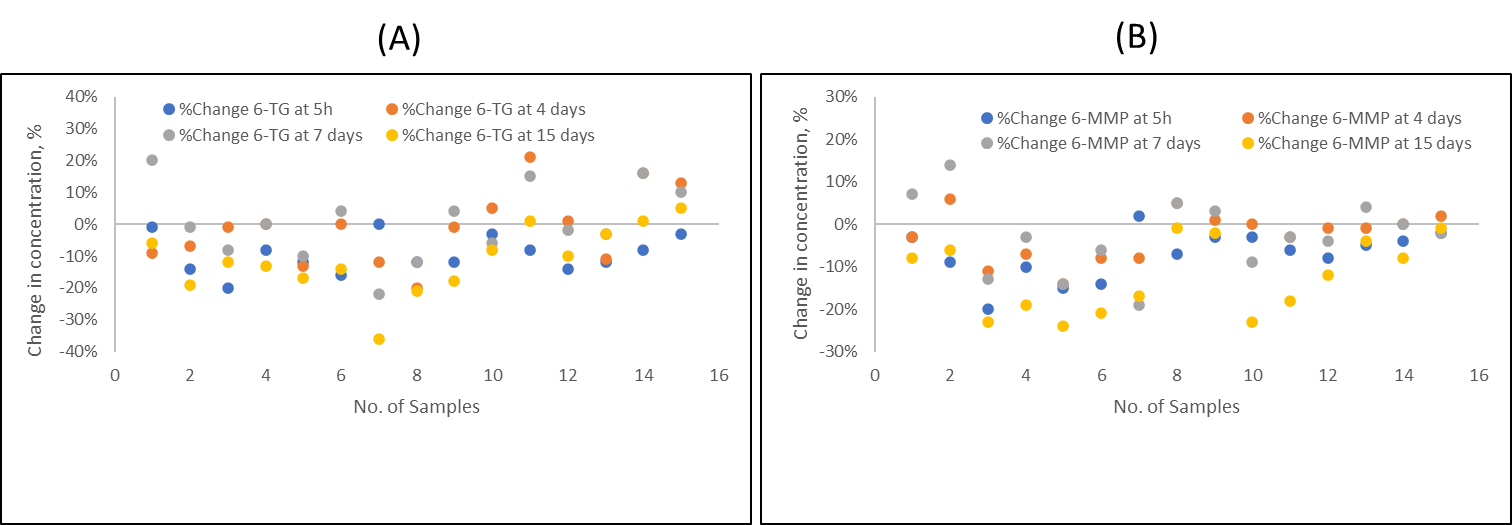
*

*Supplementary Figure S8. 6-TG and 6-MMP stability in WB samples: change in 6-TG (A) and 6-MMP (B) concentrations in WB samples stored at 4°C.*

*Supplementary Figure S9. 6-TG and 6-MMP stability in washed RBC samples: change in concentration of 6-TG and 6-MMP in processed samples stored at -70°C for 5 months (as compared to Day 1).*

*Supplementary Figure S10. 6-TG and 6-MMP stability in prepared samples: change in concentration of 6-TG and 6-MMP in extracted samples stored at -20°C for two weeks (as compared to Day 1).*


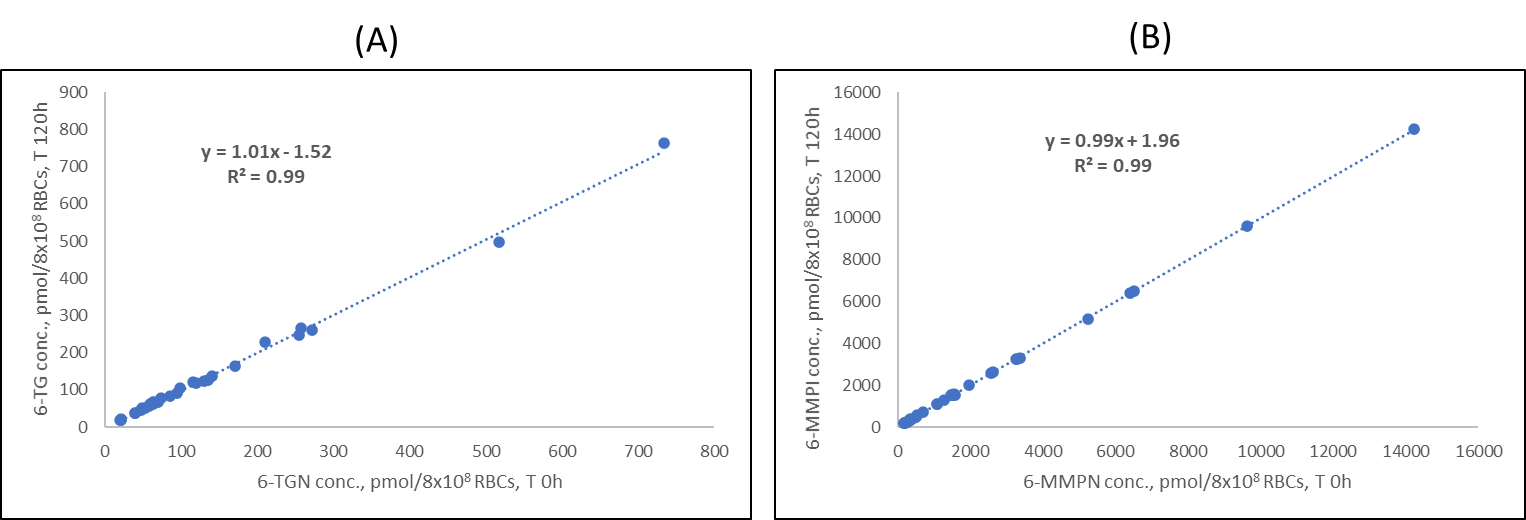


*Supplementary Figure S11. 6-TG and 6-MMP stability in the prepared samples: 6-TG (A) and 6-MMP* (B) concentration at T 120h as compared to T 0h when kept standing in autosampler at 4°C.*


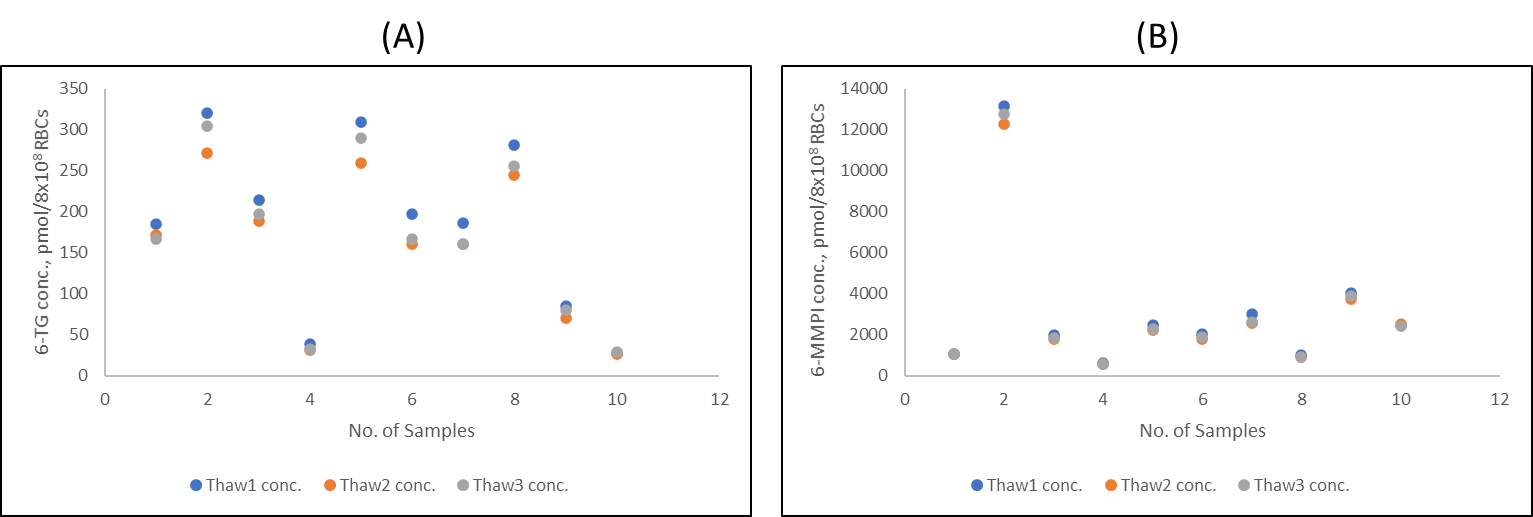


*Supplementary Figure S12. Effect of freeze-thaw cycles on 6-TG and 6-MMP stability in washed RBC samples: 6-TG (A) and 6-MMP* (B) concentration after three freeze-thaw.*

*
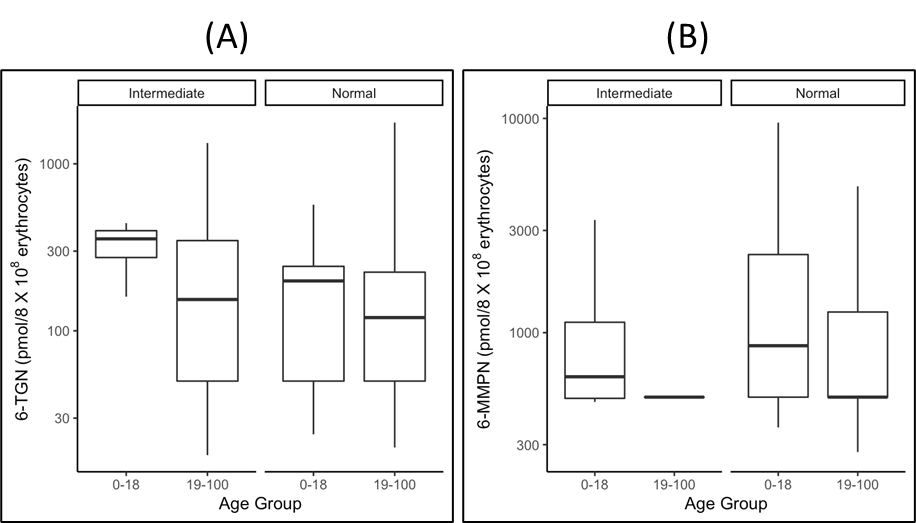
*

*Supplementary Figure S13. Distribution of 6-TG (A), and 6-MMP (B) concentrations in patient samples by age group, in patient samples with normal [6-TG (p=0.147), 6-MMP (p=0.015)] and intermediate [6-TG (p=0.161), 6-MMP* (p=0.314)] TPMT activity.*

*Supplementary Table S1 - Source parameters used in the method.*

| **Source Parameters** | **Specifics** |
| --- | --- |
| Gas Temp | 180 °C |
| Gas Flow | 7 L/min |
| Nebulizer | 40 psi |
| Sheath Gas Temp | 350 °C |
| Sheath Gas Flow | 12 L/min |
| Capillary voltage | +1500 V |
| Nozzle voltage | 0 V |

*Supplementary Table S2 - Mass transitions and instrument settings used in the method.*

| **Analyte** | **MRM transition**  **(m/z)** | **Fragmentor (V)** | **Collision energy (V)** |
| --- | --- | --- | --- |
| 6TG Quant | 168>151 | 120 | 22 |
| 6TG Qual | 168>134 | 120 | 22 |
| 6TG IS Quant | 171>154 | 120 | 22 |
| 6TG IS Qual | 171>137 | 120 | 22 |
| 6-MMP* Quant | 159>111 | 65 | 10 |
| 6-MMP* Qual | 159>83 | 65 | 30 |
| 6-MMP* IS Quant | 162>111 | 65 | 10 |
| 6-MMP* IS Qual | 162>83 | 65 | 30 |

*Supplementary Table S3 - Effect of the decanting centrifugal force on RBCs recovery. Triplicate measurements were performed for each sample.*

| No. | Decant RCF | Residual volume in the tube (µl) | RBC count (X10^6^/µ) | | | Mean RBC count (X10^6^/µl) | Standard deviation | %CV |
| --- | --- | --- | --- | --- | --- | --- | --- | --- |
| 1 | 7 | 2500 | 1.36 | 1.32 | 1.37 | 1.35 | 0.022 | 1.61% |
| 2 | 9 | 1400 | 2.06 | 2.09 | 2.12 | 2.11 | 0.024 | 1.16% |
| 3 | 11 | 500 | 3.36 | 3.25 | 3.33 | 3.29 | 0.046 | 1.41% |
| 4 | 13 | 300 | 3.43 | 3.52 | 3.18 | 3.35 | 0.144 | 4.29% |
| 5 | 16 | 200 | 2.51 | 2.44 | 2.54 | 2.49 | 0.042 | 1.68% |

*Supplementary Table S4 - Effect of the centrifugation time on RBCs recovery. MFU is McFarland units.*

| No. | Centrifugation time (sec) | Density of supernatant after centrifugation (MFU) | | | Avg. Density of sup. | RBC count (X10^6^/ul) | | | Avg. RBC count (X10^6^/ul) | STDEV | %CV |
| --- | --- | --- | --- | --- | --- | --- | --- | --- | --- | --- | --- |
| 1 | 45 | >4.0 | >4.0 | >4.0 | >4.0 | 2.35 | 2.45 | 2.11 | 2.28 | 0.143 | 6.26% |
| 2 | 120 | 2.03 | 2.56 | 2.2 | 2.26 | 2.99 | 3.12 | 2.88 | 3.00 | 0.098 | 3.27% |
| 3 | 180 | 1.52 | 1.78 | 1.65 | 1.65 | 3.17 | 3.32 | 3.07 | 3.20 | 0.103 | 3.22% |
| 4 | 240 | 0.78 | 0.81 | 0.87 | 0.82 | 3.4 | 3.41 | 3.27 | 3.34 | 0.064 | 1.91% |
| 5 | 360 | 0.49 | 0.91 | 0.85 | 0.75 | 3.35 | 3.45 | 3.01 | 3.23 | 0.188 | 5.83% |

*Supplementary Table S5 - The within-run between-run and total imprecision for analysis of RBC samples containing low and high concentrations of 6-TGRib and 6-MMPRib, and pool of WB samples from patients on thiopurine drug therapy.*

| **Sample Type** | **Within-run % CV** | | **Between-run/Day % CV** | | **Total % CV** | |
| --- | --- | --- | --- | --- | --- | --- |
|  | **6-TG** | **6-MMP*** | **6-TG** | **6-MMP*** | **6-TG** | **6-MMP*** |
| **Low** | 2.71 | 0.9 | 1.26 | 0.46 | 2.99 | 1.01 |
| **High** | 2.75 | 0.82 | 0.21 | 0.87 | 2.76 | 1.19 |
| **Positive patient samples** | 2.84 | 0.81 | 0.46 | 0.64 | 2.88 | 1.03 |

*Supplementary Table S6 - The within-run, between-run, Total imprecision and mean accuracy at the limit of quantitation of the assay for 6-TG and 6-MMP*.*

| **Expected conc. (pmol/100 μL)** | | **Within-run CV, %** | | **Between-run/Day CV** | | **Total CV, %** | | **Mean concentration (pmol/100 μL)** | | **Mean Accuracy, %** | |
| --- | --- | --- | --- | --- | --- | --- | --- | --- | --- | --- | --- |
| **6-TG** | **6-MMP*** | **6-TG** | **6-MMP*** | **6-TG** | **6-MMP*** | **6-TG** | **6-MMP*** | **6-TG** | **6-MMP*** | **6-TG** | **6-MMP*** |
| 20* | 400* | 4.76 | 1.68 | 5.65 | 1.60 | 7.39 | 2.32 | 21.8 | 405 | 109% | 101% |
| 20** | 400** | 3.94 | 2.43 | na | na | na | na | 21.4 | 388 | 107% | 96.9% |

** samples analyzed in 5 replicates on 4 days*

*** samples analyzed in 10 replicates on one day*

*Supplementary Table S7 - Common drugs evaluated for potential interference with the assay.*

| Description | Final spiked conc. μg/mL (1:20 dilution) | Interference  Y/N |
| --- | --- | --- |
| Alpha-Hydroxymidazolam | 0.5 | N |
| Buprenorphine | 0.125 | N |
| Codeine | 1 | N |
| Fentanyl | 0.05 | N |
| Meperidine | 1.25 | N |
| Midazolam | 0.5 | N |
| Morphine | 0.5 | N |
| Naloxone | 2.5 | N |
| Norfentanyl oxalate | 0.05 | N |
| Oxycodone | 1 | N |
| Oxymorphone | 1 | N |
| Zolpidem | 0.5 | N |
| (±)-Amphetamine | 1.25 | N |
| Methylphenidate | 2.5 | N |
| Norhydrocodone HCl | 2.5 | N |
| Ritalinic Acid | 10 | N |
| Tapentadol HCl | 2.5 | N |
| (±)-3,4-Methylenedioxyamphetamine | 5 | N |
| (±)-3,4-Methylenedioxymethamphetamine | 5 | N |
| (±)-Methamphetamine | 5 | N |
| Methylenedioxyethylamphetamine | 5 | N |
| Norbuprenorphine | 0.5 | N |
| 6-acetylmorphine | 0.5 | N |
| 7-Aminoclonazepam | 1 | N |
| Alpha-Hydroxyalprazolam | 0.5 | N |
| Alprazolam | 1 | N |
| Clonazepam | 0.5 | N |
| Diazepam | 1.25 | N |
| Hydrocodone | 1 | N |
| Hydromorphone | 0.5 | N |
| Nordiazepam | 1.25 | N |
| Oxazepam | 1.25 | N |
| Temazepam | 1.25 | N |
| Lorazepam | 1.5 | N |
| Normeperidine | 1.25 | N |
| Noroxycodone | 2.5 | N |
| Noroxymorphone hydrochloride | 2.5 | N |
| Phentermine | 2.5 | N |
| Gabapentin | 2.5 | N |
| Pregabalin | 2.5 | N |
| Zolpidem Phenyl-4-carboxylic acid | 2.5 | N |
| Acetaminophen | 5 | N |
| Caffeine | 5 | N |
| Chlorpheniramine | 5 | N |
| Ibuprofen | 5 | N |
| Naproxen | 5 | N |
| Pseudoephedrine | 5 | N |

*Supplementary Table S8 – Evaluation of the sample’s dilution integrity.*

| Expected concentration, pmol/8X10e8 RBC | Sample ID, dilution, replicate | Observed concentration, pmol/8X10e8 RBC | Mean concentration, pmol/8X10e8 RBC | Standard deviation | CV, % | Concentration normalized for dilution, pmol/8X10e8 RBC | Accuracy, % |
| --- | --- | --- | --- | --- | --- | --- | --- |
| 6-TGN | | | | | | | |
| 355 | Sample 1, dilution x5 1 | 77 | 80 | 2.89 | 4% | 400 | 113% |
|  | Sample 1, dilution x5 2 | 82 |  |  |  |  |  |
|  | Sample 1, dilution x5 3 | 82 |  |  |  |  |  |
|  | Sample 1, dilution x10 1 | 34 | 37 | 5.2 | 14% | 370 | 104% |
|  | Sample 1, dilution x10 2 | 34 |  |  |  |  |  |
|  | Sample 1, dilution x10 3 | 43 |  |  |  |  |  |
| 324 | Sample 2, dilution x5 1 | 71 | 73 | 2.89 | 4% | 365 | 113% |
|  | Sample 2, dilution x5 2 | 71 |  |  |  |  |  |
|  | Sample 2, dilution x5 3 | 76 |  |  |  |  |  |
|  | Sample 2, dilution x10 1 | 35 | 32 | 2.89 | 9% | 320 | 99% |
|  | Sample 2, dilution x10 2 | 30 |  |  |  |  |  |
|  | Sample 2, dilution x10 3 | 30 |  |  |  |  |  |
| 286 | Sample 3, dilution x5 1 | 59 | 62 | 2.31 | 4% | 310 | 108% |
|  | Sample 3, dilution x5 2 | 63 |  |  |  |  |  |
|  | Sample 3, dilution x5 3 | 63 |  |  |  |  |  |
|  | Sample 3, dilution x10 1 | 25 | 26 | 2.31 | 9% | 260 | 91% |
|  | Sample 3, dilution x10 2 | 25 |  |  |  |  |  |
|  | Sample 3, dilution x10 3 | 29 |  |  |  |  |  |
| 6-MMPN | | | | | | | |
| 25040 | Sample 1, dilution x5 1 | 5472 | 5552 | 94.32 | 2% | 27760 | 111% |
|  | Sample 1, dilution x5 2 | 5656 |  |  |  |  |  |
|  | Sample 1, dilution x5 3 | 5528 |  |  |  |  |  |
|  | Sample 1, dilution x10 1 | 2480 | 2,528 | 41.57 | 2% | 25280 | 101% |
|  | Sample 1, dilution x10 2 | 2552 |  |  |  |  |  |
|  | Sample 1, dilution x10 3 | 2552 |  |  |  |  |  |
| 12,720 | Sample 2, dilution x5 1 | 2616 | 2,619 | 20.13 | 1% | 13095 | 103% |
|  | Sample 2, dilution x5 2 | 2640 |  |  |  |  |  |
|  | Sample 2, dilution x5 3 | 2600 |  |  |  |  |  |
|  | Sample 2, dilution x10 1 | 1128 | 1,152 | 24 | 2% | 11520 | 91% |
|  | Sample 2, dilution x10 2 | 1176 |  |  |  |  |  |
|  | Sample 2, dilution x10 3 | 1152 |  |  |  |  |  |
| 41,440 | Sample 3, dilution x5 1 | 9520 | 9,707 | 166.53 | 2% | 48535 | 117% |
|  | Sample 3, dilution x5 2 | 9840 |  |  |  |  |  |
|  | Sample 3, dilution x5 3 | 9760 |  |  |  |  |  |
|  | Sample 3, dilution x10 1 | 4456 | 4,576 | 104 | 2% | 45760 | 110% |
|  | Sample 3, dilution x10 2 | 4640 |  |  |  |  |  |
|  | Sample 3, dilution x10 3 | 4632 |  |  |  |  |  |
| 18,560 | Sample 4, dilution x5 1 | 4072 | 3,997 | 76.04 | 2% | 19985 | 108% |
|  | Sample 4, dilution x5 2 | 4000 |  |  |  |  |  |
|  | Sample 4, dilution x5 3 | 3920 |  |  |  |  |  |
|  | Sample 4, dilution x10 1 | 1952 | 1,909 | 37.81 | 2% | 19090 | 103% |
|  | Sample 4, dilution x10 2 | 1896 |  |  |  |  |  |
|  | Sample 4, dilution x10 3 | 1880 |  |  |  |  |  |
| 23,760 | Sample 5, dilution x5 1 | 5016 | 4,944 | 92.26 | 2% | 24720 | 104% |
|  | Sample 5, dilution x5 2 | 4976 |  |  |  |  |  |
|  | Sample 5, dilution x5 3 | 4840 |  |  |  |  |  |
|  | Sample 5, dilution x10 1 | 2744 | 2,640 | 128.75 | 5% | 26400 | 111% |
|  | Sample 5, dilution x10 2 | 2680 |  |  |  |  |  |
|  | Sample 5, dilution x10 3 | 2496 |  |  |  |  |  |
